# Humanoid Robot Walking in Maze Controlled by SSVEP-BCI Based on Augmented Reality Stimulus

**DOI:** 10.3389/fnhum.2022.908050

**Published:** 2022-07-14

**Authors:** Shangen Zhang, Xiaorong Gao, Xiaogang Chen

**Affiliations:** ^1^School of Computer and Communication Engineering, University of Science and Technology Beijing, Beijing, China; ^2^Department of Biomedical Engineering, School of Medicine, Tsinghua University, Beijing, China; ^3^Institute of Biomedical Engineering, Chinese Academy of Medical Sciences and Peking Union Medical College, Tianjin, China

**Keywords:** steady-state visual evoked potential (SSVEP), based brain-computer interface (BCI), augmented reality (AR), humanoid robot, maze

## Abstract

The application study of robot control based brain-computer interface (BCI) not only helps to promote the practicality of BCI but also helps to promote the advancement of robot technology, which is of great significance. Among the many obstacles, the importability of the stimulator brings much inconvenience to the robot control task. In this study, augmented reality (AR) technology was employed as the visual stimulator of steady-state visual evoked potential (SSVEP)-BCI and the robot walking experiment in the maze was designed to testify the applicability of the AR-BCI system. The online experiment was designed to complete the robot maze walking task and the robot walking commands were sent out by BCI system, in which human intentions were decoded by Filter Bank Canonical Correlation Analysis (FBCCA) algorithm. The results showed that all the 12 subjects could complete the robot walking task in the maze, which verified the feasibility of the AR-SSVEP-NAO system. This study provided an application demonstration for the robot control base on brain–computer interface, and further provided a new method for the future portable BCI system.

## Introduction

Based brain-computer interface (BCI) provides rich and powerful command signals for auxiliary devices by directly decoding user intentions from brain signals in real time. Due to the advantages of the electroencephalography (EEG) method in time resolution, brain–computer interface technology mostly adopted EEG to realize the real-time decoding of brain intentions. In the past two decades, researchers have attempted to improve the performance of BCIs from the improvements in experimental paradigms and decoding algorithms (Chen et al., 2015b; Coogan and He, 2018; Zhang et al., 2018, 2021; Abiri et al., 2019; Rashid et al., 2020; Gao et al., 2021; Stieger et al., 2021). At present, the experimental paradigms of BCIs mainly contained motor imagination (MI), P300, and steady-state visual evoked potential (SSVEP). Among them, SSVEP-BCI was favored due to its higher signal-to-noise ratio (SNR), larger number of targets, better information transfer rate (ITR), and less training. In typical SSVEP-BCIs, decoding methods were employed to identify the frequency of SSVEP to determine which target the subject was focusing on (Li et al., 2015; Nakanishi et al., 2018; Perdikis and Millan, 2020).

Robot controlling is one of the significant application fields of BCI. Through the analysis of brain signals, human intentions could be accurately converted into robot control instructions in real-time, so as to achieve precise control of the robot (Wolpaw et al., 2002; Li et al., 2015). With the maturity of BCI technology, BCI-based robot control technology has made certain progress in theory and practice, which has promoted the practical application of BCI. Specifically, the application prospects of BCI technologies in robot control have been proven by studies on the control of wheelchairs, aircraft with four limbs, manipulators, exoskeletons, and humanoid robots based on BCIs. For example, Mahmood used convolutional neural networks to classify the evoked SSVEPs and realized the control of electric wheelchairs (Muller et al., 2013). Chen proposed a robotic arm control system that combined computer vision and BCI to realize the automatically grabs objects by robotic arm (Chen et al., 2018).

Among the many types of robots that may be improved by BCI technology, the humanoid robot has its unique advantages. Integrating a variety of sensors, humanoid robot was an intelligent robot with a human-like appearance and capable of simulating human behaviors. Due to their high intelligence, the application researches of BCI-based humanoid robots were of great significance and received increasing attention. For example, a hybrid BCI system was designed in which the robot walking instruments were controlled by SSVEP, and the robot grabbing instruments was controlled by MI (Duan et al., 2017). Another BCI system was used to control the robot to grab a glass of water so as to help patients (Spataro et al., 2017). Unfortunately, most of the previous studies were based on the traditional BCI systems which depended on a fixed visual stimulus.

Although notable progresses have been made in the study of BCI-based robot control, there are still many obstacles restricting its practical applications, one of which was the importability of the visual stimulators. The typical BCI system traditionally employed a liquid crystal display (LCD) screen to display visual stimuli, which required the subjects to switch their focus between the visual stimuli and the environment. The importability of the visual stimulation devices led to the inconvenience for the robot controlling, which required the improvement of a stimulator in portability and intelligence. Fortunately, augmented reality (AR) technology provided a new visual stimuli mode in the virtual scenes, which could be used to improve the convenience and practicability of the BCI system for robot controlling (Appaia et al., 2021; Hsu et al., 2021). At present, the studies of BCIs based on AR stimulators are in its infancy, and the feasibility of this scheme has been verified by some experiments. For example, an AR-BCI system was designed and realized the controlling of desk lamp (Kansaku et al., 2010). By combining augmented reality and SSVEP-BCI technologies, the complex navigation task of the robot was completed with more intuitive and effective interaction (Faller et al., 2017). However, the researches on BCI based on AR stimulator are in the ascendant, and further research are urgently needed.

In view of the shortcomings of previous studies, we built an AR-BCI system to explore the portability of BCI stimulus. In the AR-BCI system, SSVEP visual stimulus was projected into the virtual visual interface. We drew lessons from the previous studies of BCIs based on AR stimulus and further optimized our study. For example, scholars achieved the control of desk lamps or television by an AR-BCI system, in which BCI commands were converted into visual stimulation control panels and presented in AR (Takano et al., 2011). In another study, the realistic environment captured by the camera and visual stimulation of SSVEP were mapped to the head mounted displayer of AR for the peripheral equipment controlling (Horii et al., 2015). The combination of SSVEP visual stimulation and AR technology realized the control of robot complex navigation tasks, which achieved the more intuitive and efficient interaction between humans and computers (Faller et al., 2017). These studies verified the feasibility of AR for presenting BCI stimulation, while the more complex application studies such as the combination of AR, BCI, and robot controlling need to be further explored.

In this study, an AR-SSVEP-NAO system was designed to explore the application potentials of BCIs. The AR-SSVEP system was used to improve the importability problem of a stimulator in BCIs, in which AR provided visual stimulation of SSVEP and presented a virtual visual stimulation interface to the subjects, and the EEG signals were collected and converted into instructions to control external devices. The reliability of the AR-BCI system was verified by the performance of the introduced intelligent humanoid robot. Subjects were required to control the external equipment (Nao robot) to the complete complex walking task in the maze by using the designed AR-SSVEP-NAO system, and the system performance was verified by the online experiments.

Another feature of this study was the construction of a brain–computer interface control robot system based on a shared control strategy. In the brain-controlled robot system, according to the degree of interaction between humans and machines, there were two main typical interaction modes for brain-computer interface control systems: direct control and shared control. The system structure of direct control was relatively simple, and the EEG signal obtained by the brain-computer interface was used to directly control the robot. In face of complex control tasks, direct control required frequent control operations, which brought great mental pressure to users. While shared control integrated human and computer intelligence to better control robots. In this study, a robot system was constructed based on the shared control strategy, in which the brain-computer interface decoded the SSVEP signal to obtain control instructions, and combined the control instructions owned by the robot intelligence (with the help of MARKs) to perform control fusion, so as to achieve robot control by using fewer instruments.

## Materials and Methods

### Subjects

Twelve healthy subjects (4 females; aged 21–27 years) with normal or corrected-to-normal vision were recruited as participants in this study. Each of them attended both the random prompt and autonomous selection experiments. Before the experiment, each subject was required to understand the experimental contents and sign an informed consent form and received a monetary compensation for his or her participation. This study was approved by the Research Ethics Committee of Tsinghua University (Jan 3, 2019).

### Visual Stimuli in Augmented Reality

During the experiments, the visual stimuli were presented by the AR device of HoloLens. HoloLens was a glass of Microsoft with the function of wireless head-mounted augmented reality. With the customized dedicated chip of holographic processing unit (HPU), the AR glass could realize the combination of the real and virtual environment, thus helping the users to enter a peculiar world environment.

Developed by Unity3D and Visual Studio 2017, the visual stimuli in AR were fixed in the central visual field of subjects so as to facilitate the simultaneous observation of stimulation and experimental scene. Six targets were presented in the visual stimuli interface with the stimuli frequencies of 8 Hz (“L1”), 9.5 Hz (“S2”), 11 Hz (“R3”), 8.5 Hz (“Le4”), 10 Hz (“St5”) and 11.5 Hz (“Ri6”), respectively. The refresh rate of the HoloLens used for stimulus representation was 60Hz. This study used the sampled sinusoidal stimulation method (Manyakov et al., 2013; Chen et al., 2015b) to present visual flickers in the AR glasses. In the autonomous selection experiment, each target in the visual stimuli referred to a pre-defined robot operation instruction. Specifically, “L1,” “S2,” and “R3” represented the recognition and tracking of mark1 and then turn left, the recognition and tracking of mark2 and then stop, the recognition and tracking of mark3 and then turn right, respectively. While “Le4,” “Ri6,” and “St5” represented a 45° left turn, a 45° right turn, and stop 2 s, respectively. When one of the three marks was within the camera view, the robot would automatically recognize the mark and move toward the mark. When approaching the mark, the robot would turn or stop according to the recognized mark. When there was no mark in front of the robot, the command “L1,” “S2,” or “R3” would rotate the robot”s head camera to help find the corresponding mark. Additionally, subjects could also adjust the robot position by the command “Le4” or “Ri6.”

**FIGURE 1:**
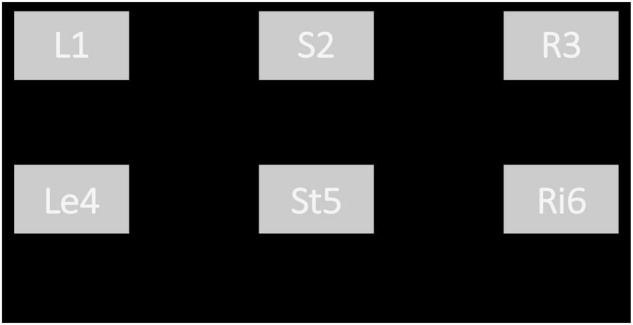
The spatial distributions of augmented reality stimulus.

The spatial distributions of the six stimuli blocks on the visual stimuli interface are shown in Figure 1. The six stimuli targets were arranged into two rows, and each row contained 3 stimuli blocks. Each stimulus was 240 × 160 pixels, the horizontal interval of the stimulus interface was 290 pixels, and the vertical interval was 200 pixels. In this study, the visual stimulus interface was presented in the front of the robot environment, so as to provide convenience for online controlling of the robot.

### Data Acquisition

EEG data were acquired by the Neuracle EEG Recorder (Neuracle, Inc.), and the sampling rate was 1,000 Hz. The nine electrodes (Pz, PO5, PO3, POz, PO4, PO6, O1, Oz, and O2) in the occipital region were selected for the EEG data recording, with the reference electrode located at the vertex. All electrodes were placed in accordance with the international 10–20 system. All the electrode impedances were lower than 10 kΩ. The recorded EEG data were sent to the PC by the WIFI of a wireless amplifier. The Neuracle EEG amplifier adopted a multi-parameter synchronizer (Neuracle, China) to synchronize EEG data to the experimental tasks. The multi-parameter synchronizer had a built-in-light port to receive triggers. Event triggers generated by the stimulus program were sent from the multi-parameter synchronizer to the EEG amplifier and recorded on an event channel synchronized to the EEG data. To ensure the frequency stability of the flicker stimulus in AR, the flicker stimulus was presented continuously throughout the process in this study. The stimulus program informed subjects to fixate on the flickering stimulus target via auditory cues while simultaneously sending event triggers. According to the event trigger, we could mark the stimulus onsets, making it easy to synchronize the EEG data with the visual stimuli. After 2-s stimulus time, a reminder of “Di” sound informed subjects to rest. The HoloLens was only used to present visual stimuli.

### NAO Robot System

The humanoid robot NAO was adopted as the equipment for the BCI controlling. The body of the NAO had 25 freedoms of movement, three touch sensors, and two cameras on the head and mouth. The two cameras could not be called at the same time for the monocular vision of NAO. In this study, an autonomous selection task was designed for the robot controlling, in which the robot should track the target mark selected by the BCI system.

**FIGURE 2:**
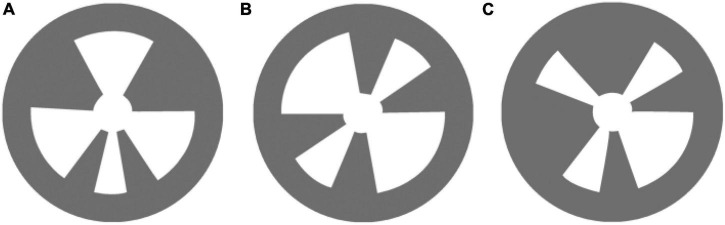
NAOMarks. **(A)** Turn left. **(B)** Turn right. **(C)** Stop.

Figure 2 shows the three NAOMarks of “turn left” (a), “turn right” (b) and “stop” (c), which were used for robot walking in this study. NAOMarks were the specific landmarks with specific patterns, which could be recognized by the robot using the vision module of ALLandMarkDetection. The landmarks could be placed at different locations in the field of robot action. The specific location of the different triangle fans was used to distinguish one Naomark from the others. Depending on which landmark was detected, the information of the robot location could be obtained.

**FIGURE 3:**
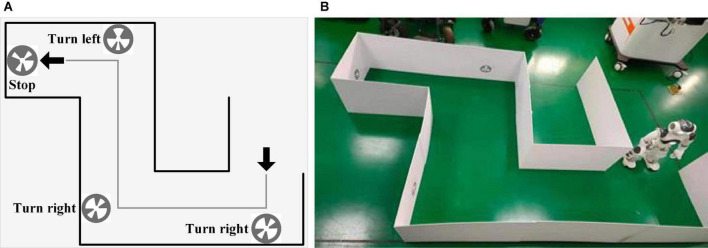
The experimental scene. **(A)** Spatial distribution of the maze. **(B)** Experimental environment.

Figure 3 shows the experimental scene in the autonomous selection experiment, including the spatial distribution (a) and the experimental environment of the maze (b). The experimental environment was a maze region surrounded by polyvinyl chloride boards (PCB), and the ground was flat and free of debris. The robot was controlled to walk in the maze, and the execution commands were prompted by the SSVEP-BCI system. The NAO robot was initially placed at the entrance of the maze and was controlled to walk and approach the end of the maze. NAOMarks were affixed at the corners of the maze. Three marks with the instructions of “Turn left,” “Turn right,” and “Stop” were employed in the robot walking experiment, as shown in Figure 2. Marks were recognized by the tool of “LandmarkTest” which is integrated into the internal API of NAO. In the process of mark recognition, the parameters of the mark (e.g., the size and deflection angle) were also obtained by “LandmarkTest.” The angle and distance between the robot and marks were calculated according to the deflection angle and mark size. The robot rotated its angle until it was directly in front of the mark, adjusted the walking pace to approach the marks, and executed the robot commands related to the MarkID. With NAOMarks that encapsulate the exact meaning of a robot”s actions, it has the potential to allow robots to perform complex tasks by using fewer control commands, eliminating the need to control specific movements through step-by-step fine-tuning as in traditional control methods.

### System Communications

A local area network (LAN) was established to realize the communication between the wireless amplifier, PC, NAO, and synchronizer. The data acquisition system was developed with MATLAB and the NAO system was developed with python, and they both worked on the same computer. Firstly, EEGs were processed by MATLAB, and then the processing results were sent to Python and further transmitted to the robot instruments. In the implementation of the robot walking task, parameters such as the running time, transmitted data, and were recorded by Python.

### Data Analysis

EEG data were firstly down-sampled 4 times (250Hz), and then band-pass filtering was performed in the range of [1 100] Hz. Filter Bank Canonical Correlation Analysis (FBCCA) was adapted to realize the identification of different targets. In the algorithm of FBCCA, filter banks were used to decompose SSVEP into sub-band components. For each component, the conventional CCA analysis was performed (Chen et al., 2015a). In the implementation of FBCCA, the delay of 140ms was considered according to the latency delay in the visual system (Chen et al., 2015a,b).

### Experimental Settings

The online experiment included the systems of AR, BCI, and NAO. The equipment used included: AR glasses, 64-lead EEG cap, Neuracle wireless amplifier, wireless router, synchronizer, and Window10 laptop. All the equipment worked on the same LAN. The AR glasses were used as a visual stimulator to evoke EEG. The EEG cap was used to collect EEG data. The wireless amplifier amplified and transmitted EEG data to the PC.

**FIGURE 4:**
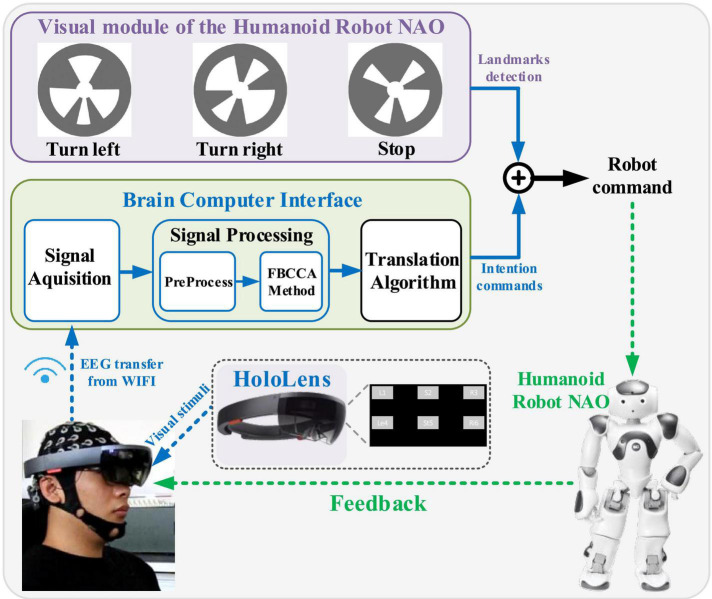
Subjects wired augmented reality equipment for the controlling of a humanoid robot.

Subjects should arm the AR equipment to complete the online experiment (Figure 4), in which the stimuli interfaces contained 6 targets, as shown in Figure 1. The brightness of the stimulation interface was adjusted to the maximum to reduce the influence of light in the environment.

The online experiment was divided into two parts: the random prompt experiment and the autonomous selection experiment, in which the robot was not involved and involved, respectively. The time length of visual stimuli was set to 2 s.

The aim of the random prompt experiment was to evaluate the performance of the proposed BCI. In the random prompt experiment, subjects should focus on the visual stimuli interface in Figure 1 that presented in augmented reality, meanwhile, PC gave the prompt sounds (“1,” “2,” “3,” “4,” “5,” “6”) randomly to remind subjects which target to gaze. After the voice prompt, there was a response time and last 0.5 s. At the end of the SSVEP stimuli, there will be a reminder of “Di” sound. If the subject made the right choice, there would be a feedback sound of “Da.” While if the wrong choice were made, there would be no feedback sound, and the subjects had been told the exact meaning of the feedback sound before the experiments. Each block contained 6 trials, and each target appeared once randomly in a block. Each trial lasted 5 s, including rest time (3 s), and stimulation time (2 s). The random prompt experiment contained 6 blocks, thus a total of 36 trials were involved.

**FIGURE 5:**
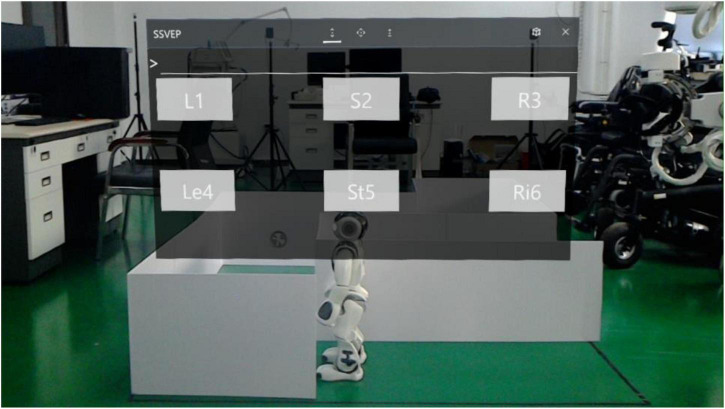
Augmented reality controlling perspective.

The autonomous selection experiment was used to evaluate the total system performance. In the autonomous selection experiment, subjects controlled the NAO robot to walk in the maze until reaching the destination, as shown in Figure 5. The virtual interface was combined with the real environment, and subjects did not need to constantly adjust the head to observe the environment and interface. Subjects were required to control the robot walking in the maze and reach the end of the maze as soon as possible. The sound “1” was used to prompt the subjects to look at the flashing interface. After a 2-s stimulus time, a “Di” sound was provided to inform subjects to rest. The robot immediately executed corresponding commands according to the recognition result of the EEG data. After the robot completed the command, the robot would send a command to the PC. Subsequently, the auditory cue for the next trial appeared. Different from the random prompt experiment, there was no auditory feedback in the autonomous selection experiment since the subjects were able to directly observe the robot”s movements. In addition, they were allowed to decide the next command independently according to the situation of the robot walking. Therefore, by using the tools of NAOMarks that encapsulate the exact meaning of a robot”s actions, the robot was allowed to perform complex tasks by using fewer control commands, eliminating the need to control specific movements through step-by-step fine-tuning as in traditional control methods. Each subject was required to complete a total of 6 blocks of the experiment, and the system running time and the detailed instructions were recorded by PC terminal.

## Results

**FIGURE 6:**
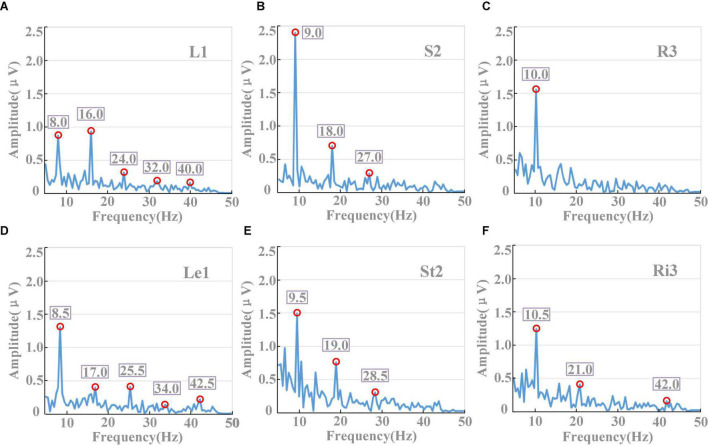
Spectra diagram from a subject (PO3). **(A–F)** Corresponded the targets of L1, S2, R3, Le1, St2, and Ri3, respectively.

Figure 6 displays the spectra diagram from a subject in the random prompt experiment. EEG data of PO3 were firstly averaged in the different stimuli conditions, respectively. Then, the averaged EEG data were processed by FFT. The results showed that in each of the conditions with different stimuli frequencies, the spectra diagrams showed obvious amplitudes in fundamental, first/second harmonic frequencies. For example, for the target of “L1” with the stimuli frequency of 8Hz (Figure 6A), the amplitudes were 0.86, 0.56, 0.39 μV for the frequencies of 8, 16, and 24 Hz, respectively. Similar conclusions could also be inferred from other conditions. The result of Figure 6 shows the reliability of stimulation and indicated that the tool of AR could be used as an effective stimulus for the application of brain–computer interface.

**FIGURE 7:**
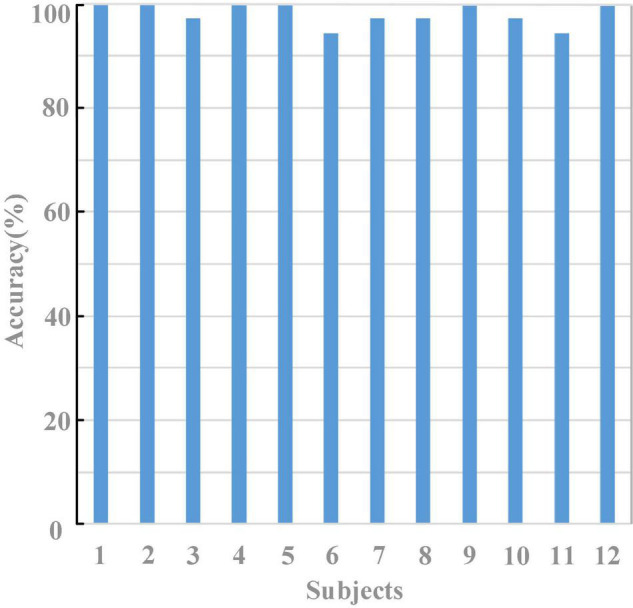
The averaged accuracies in a random prompt experiment.

Figure 7 shows the results of the random prompt experiment for all the 12 subjects. Excellent performance has been achieved, and the average accuracy of the 12 subjects was 98.15 ± 2.07%. Specifically, six subjects achieved an accuracy of 100% among the 12 subjects, and no subject achieved an accuracy lower than 90%.

**FIGURE 8:**
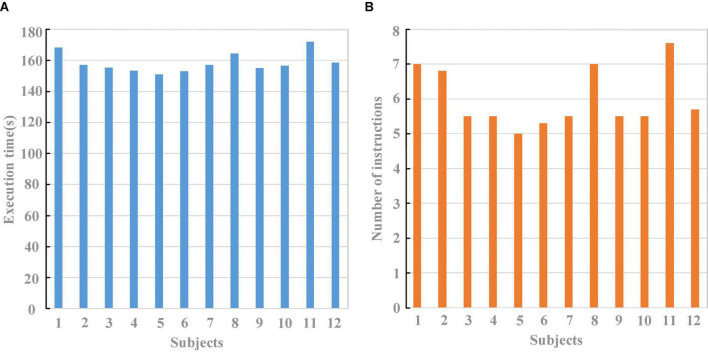
The results of autonomous selection experiment. **(A)** Execution time. **(B)** Number of instructions.

Figure 8 shows the results of the autonomous selection experiment. The system execution time contained the time of the BCI system and the execution time of the robot in the maze walking task. The time of the BCI system included the visual stimuli time (2 s) and rest time (3 s). The average execution time was 158.6 ± 5.9 s and the average number of instructions was 6.0 ± 0.79. The execution time in some blocks was larger than that of others. The time of some blocks was longer in some blocks. If the target selection was wrong, subjects need to re-select the flashing target to complete the remaining tasks, resulting in the average controlling time was longer and the number of instructions being larger than in the favorable situation. The feasibility of the system was verified by the online experiment, which indicated that all the 12 subjects could control the robot to complete the maze walking task with an average of 6 instructions.

## Discussion

This study combined brain–computer interface with robot intelligence, and verified the feasibility of BCI-based robot control in performing complex tasks. BCI has been shown to enable excellent control of robotic devices by using non-SSVEP methods (Meng et al., 2016; Edelman et al., 2019; Pulferer et al., 2022), while SSVEP-BCI method is considered to have a better potential for controlling robotics due to its high information transfer rate, good signal-to-noise ratio, easy quantification, and less user training (Ng and Goh, 2020; Li and Kesavadas, 2021). In this study, we designed an online experiment in which the SSVEP-BCI system and humanoid robot were combined for the robot walking task in the maze. We attempted to apply the technology of brain–computer interface to robot control, and the results verified the feasibility of BCI for auxiliary equipment control. In fact, as done in this article, it is of great significance to carry out the application research of robot control based on brain–computer interface, which may help disabled patients use assistive equipment, and further promote the practicality of brain–computer interfaces and the advancement of robotics.

This study further improved the visual stimulator of the brain–computer interface and verified the feasibility of AR stimulation in robot control tasks. Of the many factors that restricted the progresses of brain–computer interfaces, the non-portability of the stimulator was one typical factor that limited the practical applications of BCI. To deal with the above restrictions and further promote the application of brain–computer interfaces, this study introduced the AR technology to optimize the visual stimuli mode, which provided convenience for the control of robot walking tasks. The results indicated that all subjects were able to use the AR-SSVEP-NAO stem to complete the complex robot walking task in the maze and verified the feasibility of the combination of AR technology and BCI.

In this study, a brain–computer interface control robot system was constructed based on human–machine shared control technology. Combining the characteristics of human and machine intelligence, it had obvious advantages compared to the direct control of the brain–computer interface control technology. On the one hand, the human–machine shared control system constructed in this study combined the advantages of human-level planning and machine-level fine control, and achieved good control effects. On the other hand, in the shared control constructed in this study, machine intelligence could perceive the surrounding environment, reducing the workload of the user continuously sending instructions to drive the machine equipment.

The contribution of this research was not only reflected in the improvement of the portability of BCI but more importantly, this research realized the robot control based on shared-control mode, that was to complete the more complex robot motion control based on less control instructions. Besides the portability of augmented reality-based visual stimuli, another feature of this study was the use of only a small number of stimulus targets to achieve complex robotic motion control. With the landmark tracking method of robot vision, the robot could be controlled to detect and track specific landmarks by using only one BCI command, instead of using specific robot motion commands (such as forward, backward, and rotation) to operate the robot step by step. This study enabled robots to perform complex tasks with fewer control commands by using NAOMarks that encapsulated the exact meaning of robot actions, without the need for detailed control of all tedious actions to accomplish specific complex tasks as in traditional control methods. The results of this study were expected to provide new ideas for improving the efficiency of peripheral control based on brain–computer interface.

Although good results have been achieved, the study could further be improved in the following points: First, the advantage of a robot could be further improved. In this study, the monocular vision was adopted for marks recognition, and it could be further improved as a binocular vision in which the three-dimensional coordinates of the object could be obtained so as to achieve better results of recognition. Second, the capability of AR could be improved. AR equipment could be improved not only in the anti-interference capability but also in the perspective. Third, the portability of the equipment could be optimized so as to further improve the users’ experiences. Fourth, the maze designed in this study was a relatively simple one, and we would further increase the complexity of robot tasks in future studies so as to further highlight the superiority of the NAOMarks-based tool. Fifth, the comparative analysis of AR and traditional stimulation methods could be involved in the future studies to better highlight and explain the advantages of AR-based robotic control, and the spatial distribution of the stimulus targets might also be further optimized (Kian et al., 2011). Last, supervised methods could be adopted to further improve the decoding performance.

## Conclusion

This study attempted to explore the applications of brain–computer interface in the field of robot control. To eliminate the inconvenience of traditional visual stimulators in which subjects received visual stimuli from a fixed stimulator such as an LCD screen, this study employed augmented reality technology to display the visual stimuli of SSVEP-BCI. The robot walking task in the maze was designed to verify the applicability of the AR-BCI system. Human intentions were decoded by the BCI system and were converted into robot control commands. Results of the online experiment showed that all the 12 subjects could control the robot to complete the robot walking task. This study verified the reliability of the SSVEP-NAO system and indicated the feasibility of the AR-SSVEP-NAO system in the robot walking tasks in the maze.

## Data Availability Statement

The original contributions presented in this study are included in the article/supplementary material, further inquiries can be directed to the corresponding author.

## Ethics Statement

The studies involving human participants were reviewed and approved by Institution Review Board of Tsinghua University. The patients/participants provided their written informed consent to participate in this study.

## Author Contributions

XC designed research. SZ performed research. SZ and XC analyzed data. All authors wrote the manuscript, contributed to the article, and approved the submitted version.

## Conflict of Interest

The authors declare that the research was conducted in the absence of any commercial or financial relationships that could be construed as a potential conflict of interest.

## Publisher’s Note

All claims expressed in this article are solely those of the authors and do not necessarily represent those of their affiliated organizations, or those of the publisher, the editors and the reviewers. Any product that may be evaluated in this article, or claim that may be made by its manufacturer, is not guaranteed or endorsed by the publisher.

## References

[B1] Abiri R. Borhani S. Sellers E. W. Jiang Y. Zhao X. P. (2019). A comprehensive review of EEG-based brain-computer interface paradigms. *J. Neural Eng.* 16:011001. 10.1088/1741-2552/aaf12e 30523919

[B2] Appaia P. De Benedetto E. Duraccio L. (2021). Design, implementation, and metrological characterization of a wearable, integrated AR-BCI hands-free system for health 4.0 monitoring. *Measurement* 177:109280. 10.1016/j.measurement.2021.109280

[B3] Chen X. Wang Y. Gao S. Jung T. P. Gao X. (2015a). Filter bank canonical correlation analysis for implementing a high-speed SSVEP-based brain-computer interface. *J. Neural Eng.* 12:046008. 10.1088/1741-2560/12/4/046008 26035476

[B4] Chen X. Wang Y. Nakanishi M. Gao X. Jung T. P. Gao S. (2015b). High-speed spelling with a noninvasive brain–computer interface. *Proc. Natl. Acad. Sci. U.S.A.* 112 E6058–E6067. 10.1073/pnas.1508080112 26483479 PMC4640776

[B5] Chen X. Zhao B. Wang Y. Xu S. Gao X. (2018). Control of a 7-DOF robotic arm system with an SSVEP-based BCI. *Int. J. Neural Syst.* 28:181. 10.1142/S0129065718500181 29768990

[B6] Coogan C. He B. (2018). Brain-computer interface control in a virtual reality environment and applications for the internet of things. *IEEE Access* 6 10840–10849. 10.1109/ACCESS.2018.2809453 30271700 PMC6157750

[B7] Duan F. Lin D. Li W. Zhang Z. (2017). Design of a multimodal EEG-based hybrid BCI system with visual servo module. *IEEE Trans. Auton. Ment. Dev.* 7 332–341.

[B8] Edelman B. Meng J. Suma D. Zurn C. Nagarajan E. Baxter B. (2019). Noninvasive neuroimaging enhances continuous neural tracking for robotic device control. *Sci. Robot.* 4:eaaw6844. 10.1126/scirobotics.aaw6844 31656937 PMC6814169

[B9] Faller J. Allison B. Z. Brunner C. Scherer R. Schmalstieg D. Pfurtscheller G. (2017). A feasibility study on SSVEP-based interaction with motivating and immersive virtual and augmented reality. *arXiv* [Preprint]. arXiv:1701.03981

[B10] Gao X. Wang Y. Chen X. Gao S. (2021). Interface, interaction, and intelligence in generalized brain–computer interfaces. *Trends Cogn. Sci.* 25 671–684. 34116918 10.1016/j.tics.2021.04.003

[B11] Horii S. Nakauchi S. Kitazaki M. (2015). “AR-SSVEP for brain-machine interface: estimating user’s gaze in head-mounted display with USB camera,” in *Proceedings of the 2015 IEEE Virtual Reality (VR).* (Arles: IEEE). 10.1109/VR.2015.7223361

[B12] Hsu H. T. Shyu K. K. Hsu C. C. Lee L. H. Lee P. L. (2021). Phase-approaching stimulation sequence for SSVEP-based BCI: a practical use in VR/AR HMD. *IEEE Trans. Neural Syst. Rehabil. Eng.* 29 2754–2764. 10.1109/TNSRE.2021.3131779 34847036

[B13] Kansaku K. Hata N. Takano K. (2010). My thoughts through a robot”s eyes: an augmented reality-brain-machine interface. *Neurosci. Res.* 66 219–222. 10.1016/j.neures.2009.10.006 19853630

[B14] Kian Ng Andrew B. Bradley P. Cunnington R. (2011). “Effect of competing stimuli on SSVEP-based BCI,” in *Proceedings of the 2011 Annual International Conference of the IEEE Engineering in Medicine and Biology Society.* (Boston, MA: IEEE). 10.1109/IEMBS.2011.6091556 22255780

[B15] Li W. Li M. Zhao J. (2015). Control of humanoid robot via motion-onset visual evoked potentials. *Front. Syst. Neurosci.* 8:247. 10.3389/fnsys.2014.00247 25620918 PMC4287730

[B16] Li Y. Kesavadas T. (2021). SSVEP-based brain-computer interface for part-picking robotic co-worker. *J. Comput. Inf. Sci. Eng.* 22 1–13. 10.1115/1.4051596

[B17] Manyakov N. Chumerin N. Robben A. Combaz A. van Vliet M. Van Hulle M. (2013). Sampled sinusoidal stimulation profile and multichannel fuzzy logic classification for monitorbased phase-coded SSVEP brain–computer interfacing. *J. Neural Eng.* 10:036011. 10.1088/1741-2560/10/3/036011 23594762

[B18] Meng J. Zhang S. Bekyo A. Olsoe J. Baxter B. He B. (2016). Noninvasive electroencephalogram based control of a robotic arm for reach and grasp tasks. *Sci. Rep.* 6:38565. 10.1038/srep38565 27966546 PMC5155290

[B19] Muller S. Bastos T. Sarcinelli-Filho M. (2013). Proposal of a SSVEP-BCI to command a robotic wheelchair. *J. Control Autom. Electr. Syst.* 24 97–105. 10.1007/s40313-013-0002-9

[B20] Nakanishi M. Wang Y. Chen X. Wang Y. T. Gao X. Jung T. P. (2018). Enhancing detection of SSVEPs for a high-speed brain speller using task-related component analysis. *IEEE Trans. Biomed. Eng.* 65 104–112. 28436836 10.1109/TBME.2017.2694818 PMC5783827

[B21] Ng W. K. Goh S. Y. (2020). Indirect control of an autonomous wheelchair using SSVEP BCI. *J. Robot. Mechatron.* 32 761–767.

[B22] Perdikis S. Millan J. R. (2020). Brain-machine interfaces: a tale of two learners. *IEEE Syst. Man Cybern. Mag.* 6 12–19.

[B23] Pulferer H. S. Mondini V. Sburlea A. I. (2022). Continuous 2D trajectory decoding from attempted movement: across-session performance in able-bodied and feasibility in a spinal cord injured participant. *J. Neural Eng.* 19:036005. 10.1088/1741-2552/ac689f 35443233

[B24] Rashid M. Sulaiman N. Majeed A. P. P. A. Musa R. M. Khatun S. (2020). Current status, challenges, and possible solutions of EEG-based brain-computer interface: a comprehensive review. *Front. Neurorobot.* 14:25. 10.3389/fnbot.2020.00025 32581758 PMC7283463

[B25] Spataro R. Chella A. Allison B. Giardina M. Sorbello R. Tramonte S. (2017). Reaching and grasping a glass of water by locked-in ALS patients through a BCI-controlled humanoid robot. *Front. Hum. Neurosci.* 11:68. 10.3389/fnhum.2017.00068 28298888 PMC5331030

[B26] Stieger J. R. Stephen E. Jiang H. Cline C. C. Jo K. M. He B. (2021). Mindfulness improves brain–computer interface performance by increasing control over neural activity in the alpha band. *Cereb. Cortex* 31 426–438. 10.1093/cercor/bhaa234 32965471 PMC7727383

[B27] Takano K. Hata N. Kansaku K. (2011). Towards intelligent environments: an augmented reality-brain-machine interface operated with a see-through head-mount display. *Front. Neurosci.* 5:60. 10.3389/fnins.2011.00060 21541307 PMC3082767

[B28] Wolpaw J. R. Birbaumer N. McFarland D. J. Pfurtscheller G. Vaughan T. M. (2002). Brain-computer interfaces for communication and control. *Clin. Neurophysiol.* 11 767–791. 10.1016/S1388-2457(02)00057-3 12048038

[B29] Zhang S. Chen X. Wang Y. Liu B. Gao X. (2021). Modulation of brain states on fractal and oscillatory power of EEG in brain-computer interfaces. *J. Neural Eng.* 18:056047. 10.1088/1741-2552/ac2628 34517346

[B30] Zhang S. Han X. Chen X. Wang Y. Gao S. Gao X. (2018). A study on dynamic model of steady-state visual evoked potentials. *J. Neural Eng.* 15:046010. 10.1088/1741-2552/aabb82 29616978

